# Prevalence of adolescent pregnancy and evaluation of pregnancy outcomes: a retrospective study

**DOI:** 10.1007/s00404-025-07931-w

**Published:** 2025-01-20

**Authors:** Elmin Eminov, Ayşe Eminov

**Affiliations:** 1 Faculty of Medicine, Department of Obstetrics and Gynecology, Düzce University, 81100 Düzce, Türkiye; 2 Faculty of Health Sciences, Kocaeli Health and Technology University, Kocaeli, Türkiye

**Keywords:** Adolescent pregnancy, Preterm birth, Pre-eclampsia

## Abstract

**Introduction:**

According to the World Health Organization, adolescent pregnancy is defined as pregnancies of women aged 19 and below. The study aims to analyze the rates of adolescent pregnancies and maternal and fetal outcomes among births within the hospital and compare them with adult pregnancies.

**Methods:**

The study is conducted retrospectively in one of Turkey’s socio-economically underdeveloped provinces. The study comprises 16,985 women: 1719 adolescents and 15,266 adults who gave birth in the hospital between January 1, 2020, and December 31, 2023. All data were recorded in the SPSS 28.0 program, and the Kolmogorov–Smirnov test, Chi-Square test, ANOVA, and Independent Simple T-test were applied to analyze the data.

**Results:**

In the study, the adolescent pregnancy rate is found to be 10,1%. The mean maternal age (*p* = 0.000), gravida (*p* = 0.000), parity (*p* = 0.000), and number of abortions (*p* = 0.002) are significantly higher in the adult group. No difference is found between the groups in terms of gestational age (*p* = 0.067). Newborn birth weight was significantly higher in the adult group (*p* = 0.000). Cesarean section rates are higher in the adult group (*p* = 0.001). No difference is found in terms of stillbirth rates. No difference is found between the groups in terms of pre-eclampsia (*p* = 0.792). No difference is found between the groups in terms of preterm birth (*p* = 0.664).

**Conclusion:**

In conclusion, it came out that, according to the results of the study, the rates of premature birth, pre-eclampsia, and stillbirth in adolescents and the first and fifth-minute Apgar scores are similar to adults. However, newborn birth weights are lower in the babies of adolescent pregnant women. In addition, cesarean section rates are higher in the adult group.

## What does this study add to the clinical work

**Table:** 

This study found that adolescent pregnancies were associated with lower birth weight and lower rates of cesarean delivery, but rates of prematurity, preeclampsia, and stillbirth were similar to adult pregnancies.	

## Introduction

Adolescence covers the age range of 10 to 19, and 1,2 billion people worldwide are in this age range [1]. Around the world, approximately 11% of births occur in the adolescent age group, which is a significant rate. According to World Health Organization (WHO) data, 21 million adolescent women become pregnant each year in the world, and the vast majority of these result in birth [2]. Adolescent pregnancy rates, which were 64.5% per 1000 women in 2000, have dropped to 42.5% in 2021. Although adolescent pregnancy rates are on a downward trend globally, they continue to be seen at high rates of 97.9% and 51.4% per 1000 women, respectively, in countries such as Sub-Saharan Africa and the Caribbean [2].

Adolescence is an interim period in transitioning from childhood to adulthood, including physical and psychological changes. Thanks to these changes, adolescents begin to get to know the outside world. This process of getting to know adolescents also means that they face many dangers. For example, adolescents who do not have sufficient information about sexual activity and birth control methods can get pregnant after their first sexual experience, either by their own will or after being sexually abused, and then face serious complications related to pregnancy. Lack of support from family, growing up in a low socio-economic environment, early marriage, substance abuse, sexual abuse, lack of health services, and low education levels are the main risk factors for adolescent pregnancies [2, 3].

Pregnant adolescents frequently encounter pregnancy complications such as pre-eclampsia, pregnancy-related bleeding, anemia, difficult labor, premature rupture of membranes, low birth weight, premature birth, stillbirth, and congenital anomalies [4–8]. Perhaps the majority of adolescent pregnancies are unwanted and illegitimate [9]. Considering all these, adolescent pregnancies should be considered in the risky pregnancy category.

These women fall behind in their education during pregnancy, and since they are already mostly socio-economically inadequate, they may be excluded from society. In such cases, it becomes difficult for pregnant adolescents to access health services, and these women who receive inadequate prenatal care are exposed to the complications mentioned above [10]. As a result, adolescents who do not receive sufficient education and cannot have a profession continue to be an economic burden for countries. Although the vast majority of adolescent pregnancies are seen in developing countries, they are also seen in significant numbers in developed countries. Therefore, this situation is a problem for all countries in the world. We believe that all countries should implement programs to reduce adolescent pregnancy rates and to support adolescents socio-economically and integrate them into society.

The study is aimed to determine the prevalence of adolescent pregnancies that gave birth in the hospital and to compare them with adult pregnancies in terms of maternal and fetal outcomes.

## Materials and methods

The study is conducted in one of Turkiye’s most socio-economically underdeveloped provinces. The purpose of choosing this province is that it is one of the most socio-economically underdeveloped provinces according to the Turkish Statistical Institute data and is among the top five provinces in terms of fertility rate.

The study is conducted as a retrospective cohort study. Data from pregnant women who gave birth between January 1, 2020, and December 31, 2023, are examined. The study was conducted in Ağrı Training and Research Hospital, and the study data were obtained from hospital records. The study did not include patients whose information was missing from the required data. The study is examined by overall of 16,985 patient files.

The study is conducted by two groups. The first group included pregnant adolescents, and the second included pregnant adults. The adolescent pregnancy group included 1,719 pregnant women aged 19 and under. The adult group included 15,266 pregnant women aged 20 and over. Maternal age was considered the mother’s age in completed years at the time of delivery.

Both maternal and fetal data are examined in the study. Maternal data included maternal age at birth, gravida, parity and abortion numbers, gestational weeks, live or stillbirth rates, preterm and term birth rates, normal and cesarean birth rates, and cesarean indications. In contrast, fetal data included birth weights, first and fifth-minute Apgar scores, and genders of newborns. Pregnancies of 37 completed weeks and above were considered term pregnancies, while pregnancies of less than 37 weeks are considered preterm pregnancies. Preterm births were classified into subgroups: moderate-late preterm birth, very preterm birth, and extremely preterm birth.

A pregnancy of 22 weeks or more completed according to the last menstrual period or a newborn with a birth weight of over 500 g was considered delivery and included in the study. Those who did not meet these criteria were considered as miscarriage and were not included in the study.

The primary aim of this study was to compare normal vaginal birth rates in adolescent and adult pregnancies. The secondary aims of the study were to compare parameters such as cesarean delivery, indications for cesarean delivery, preterm delivery, pre-eclampsia, stillbirth rates, and newborn birth weights in adolescents and adults.

To conduct the study, ethical approval is retrieved from Ağrı İbrahim Çeçen University Scientific Research Ethics Committee as of 31.10.2024 with code 398.

SPSS 28.0 program is used in the analysis of the data. In categorical measurements, percentage values are used, and mean and standard deviation values are used in descriptive statistics. Kolmogorov–Smirnov test and skewness-kurtosis values determine whether the variables met the normality assumption. The chi-square test is used to compare categorical and continuous variables. The One-way ANOVA test is used to measure the effect of more than one continuous independent variable on the dependent variable, and an Independent Simple T-test is used to compare two independent variables. The results are considered statistically significant at the p < 0.05 level.

## Results

Maternal and fetal comparative results of adolescent and adult pregnancies are shown in the tables below—Table 1 lists demographic data and maternal outcomes by age group. The mean age of the patients in the adolescent group is 18.23 ± 0.880, while the mean age of the adult group is 27.82 ± 5.649, and the difference between the groups is statistically significant (Table 1).

**Table 1:** Demographic data and maternal outcomes by maternal age groups

Variables		Mother age groups				Total		*P* value
		Adolescent group		Adult group				
		Mean ± SD		Mean ± SD				
Average maternal age		18.23 ± 0.880		27.82 ± 5.649		t (16.983) = − 190.125		0.000**
		*N*	%	*N*	%	*N*	%	
Gravida	Primigravida	670	39.0	3610	23.6	4280	25.2	0.000*
	Multigravida	1049	61.0	11,656	76.4	12,705	74.8	
Parity	Nulliparity	738	42.9	3995	26.2	4733	27.9	0.000*
	Multiparity	981	57.1	11,271	73.8	12,252	72.1	
Number of abortion	No abortion	1365	79.4	11,503	75.4	12,868	75.8	0.000*
	One or more abortion	354	20.6	3763	24.6	4117	24.2	
Gestational age at delivery	Extremely preterm	9	0.5	57	0.4	66	0.4	0.792*
	Very preterm	31	1.8	261	1.7	292	1.7	
	Moderate-late preterm	259	15.1	2274	14.9	2533	14.9	
	Term	1420	82.6	12,674	83.0	14,094	83.0	
Delivery type	Vaginal delivery	**1070**	**62.2**	**8895**	**58.3**	**9965**	**58.7**	0.001*
	Cesarean delivery	**649**	**37.8**	**6371**	**41.7**	**7020**	**41.3**	
Pre-eclampsia rates		47	2.7	401	2.6	448	2.6	0.792*

*Chi-square Test

**Independent Simple T-test

Gravidity, parity and abortion numbers were evaluated according to maternal age groups. While primigravida rates were higher in adolescent pregnant women, multigravida rates were higher in the adult group, and these results were statistically significant. In addition, nulliparity rates were higher in adolescent pregnant women, while multiparity rates were higher in the adult group, and the results were statistically significant. When we examined abortion numbers, the number of one or more abortions was higher in adults. (Table 1).

The groups are compared according to their mean gestational age at delivery; no statistically significant difference is found between them (Table 1).

The groups are compared according to the type of delivery. In the adolescent group, 1070 (62,2%) patients are found to have a vaginal delivery, and 649 (37,8%) patients are found to have a cesarean section. In addition, in the adult group, 8895 (58,3%) patients are found to have a vaginal delivery, and 6371 (41,7%) patients are found to have a cesarean section. The cesarean section rate is higher in the adult group, and this difference was evaluated as statistically significant (Table 1).

The groups according to pre-eclampsia rates are compared. Pre-eclampsia rates are similar in both groups; no statistical difference is observed (Table 1).

In Table 2, indications for cesarean section are classified according to age groups. Previous uterine surgery (myomectomy or previous cesarean section) is found to be the most common reason for cesarean section in both the adolescent group (43,5%) and the adult group (53,1%). In the groups, cesarean section rates are found to be significantly higher when compared with the adult group due to previous uterine surgery (Table 2).

**Table 2:** Indications for cesarean-section by age groups

Variables	Mother age groups				Total		*P* value
	19 years and under		20 years and older				
	*N*	%	*N*	%	*N*	%	
Previous uterine surgery	**282**	**43.5**	**3383**	**53.1**	**3665**	**52.2**	0.000*
Fetal distress	129	19.9	1081	17.0	1210	17.2	0.062*
Breech presentation	**65**	**10.0**	**384**	**6.0**	**449**	**6.4**	0.000*
Non-progressive labor	37	5.7	343	5.4	380	5.4	0.734*
Multiple pregnancy	31	4.8	285	4.5	316	4.5	0.723*
Hypertensive diseases of pregnancy	18	2.8	200	3.1	218	3.1	0.609*
Macrosomic fetus	**28**	**4.3**	**180**	**2.8**	**208**	**3.0**	0.033*
Cephalopelvic disproportion	6	0.9	57	0.9	63	0.9	0.939*
Other causes	53	8.1	458	7.2	511	7.3	0.488*
Total	649	100.0	6371	100.0	7020	100.0	

*Chi-square Test

When the primary cesarean section rates were examined, the most common reason in both groups came out as fetal distress. It was determined that 129 (19,9%) patients in the adolescent group and 1,081 (17,0%) patients in the adult group had a cesarean section due to fetal distress. When these rates are compared, no statistically significant difference is found (Table 2).

Breech presentation is the third most common reason for the primary cesarean section. Cesarean section rates due to breech presentation are statistically significantly higher in the adolescent group (Table 2).

The rates of cesarean section due to non-progressive labor, multiple pregnancies, hypertensive diseases of pregnancy, and cephalopelvic disproportion are similar in both groups (Table 2). Cesarean section rates due to macrosomic fetuses are significantly higher in adolescents (Table 2). Placental abruption, cord prolapse, maternal disproportion, and presentation anomalies such as forehead and face presentation are collected under other indications, and no statistical difference is found between the groups (Table 2).

Fetal outcomes are summarized in Table 3. The groups are compared according to live and stillbirth rates. It is determined that 15 patients (0,9%) in the adolescent group and 107 patients (0,7%) in the adult group have stillbirth. According to these results, no statistically significant difference is found between the groups (Table 3).

**Table 3:** Fetal outcomes by maternal age groups

Variables		Mother age groups				Total		*P* value
		19 years and under		20 years and older				
		*N*	%	*N*	%	*N*	%	
Live and stillbirth rates	Live birth	1704	99.1	15,159	99.3	16,863	99.3	0.424*
	Stillbirth	15	0.9	107	0.7	122	0.7	
Gender	Girl	875	50.9	7394	48.4	8269	48.7	0.053*
	Male	844	49.1	7871	51.6	8715	51.3	
Birth weight	Extremely low birth weight (ELBW)	13	0.8	69	0.5	82	0.5	0.000*
	Very low birth weight (VLBW)	9	0.5	92	0.6	101	0.6	
	Low birth weight (LBW)	227	13.2	1497	9.8	1724	10.2	
	2501 g and above	1470	85.5	13,608	89.1	15,078	88.8	

		Mean ± SD		Mean ± SD		*T* value		*P* value
Apgar 1st minute		7.78 ± 1.018		7.81 ± 0.971		t (16.983) = − 1.132		0.258**
Apgar 5th minute		8.83 ± 1.075		8.84 ± 1.027		t (16.983) = − 0.535		0.593**

*Chi-square Test

**Independent Simple T test

Both groups are compared according to the gender of the newborns, and no statistically significant difference is found between the groups (Table 3).

In addition, the groups are compared according to the birth weights of the newborns, and a statistically significant difference is found between the groups. While the average birth weight of the babies of the patients included in the adolescent group is 2959,84 ± 493.514 gr, the average birth weight of the babies in the adult group is found to be 3072,96 ± 512.486 gr (Table 3).

To evaluate the postpartum well-being of the newborns, the groups are compared according to the first and fifth-minute Apgar scores. No statistically significant difference is found between the groups in terms of both the first-minute Apgar scores and the fifth-minute Apgar scores (Table 3).

## Discussıon

Adolescent pregnancies are a health problem that concerns all countries in the world. The study is aimed to evaluate the results of adolescent pregnancies and adult pregnancies. The study was conducted in one of the most socio-economically underdeveloped, rural provinces of Turkiye. Despite this, it is determined that the adolescent pregnancy rate is 10,1% in the study, and this value is better than the results of many countries [11]. When the results were examined, it became quite clear that the mean maternal age, gravida, parity, and abortion numbers were significantly higher in the adult group. When this is compared according to the gestational age at delivery, there was no difference between the groups. In addition, there was no difference between the groups according to the gender of the newborns.

Cesarean section birth rates in adolescent pregnancies vary by country. It is assumed that cesarean-section birth rates may be higher in adolescents who have not yet completed their physiological and anatomical development [12]. There are studies in the literature that support this and also report the opposite. In their study, Özdemirci and colleagues found that the rate of cesarean delivery was higher in adolescent pregnant women [13]. On the contrary, Medhi et al. found no difference in cesarean section between pregnant adolescents and pregnant adults in their study [14]. Eriksen and colleagues also retrospectively examined births in Boston between 2000 and 2012 and found lower cesarean delivery rates in adolescents [15]. Similarly, a systematic review conducted in Brazil covering 2002 to 2012 reported that cesarean section rates were lower in adolescents than in adults [16]. In the study, the groups are compared according to the type of birth. It was found that the rate of cesarean births was higher in adults than in adolescents. It is thought that this may be due to the increase in the desire of women to have a cesarean birth in recent years to avoid labor pain. In addition, the number of patients who have a cesarean section for tubal ligation has become considerably more common in adult women. In addition, cesarean sections are commonly performed in adult women for reasons such as advanced maternal age, cosmetic reasons, and pelvic organ prolapse.

In the study, the groups according to cesarean indications are compared. By the study, no significant difference between the groups in terms of cesarean indications are observed. It is quite clear that the first three cesarean indications were previous uterine surgery, fetal distress, and breech presentation. It is believed that the reason why the indication for previous uterine surgery is as common among adolescents as it is among adults is that the majority of pregnant women in the adolescent group are in late adolescence. Those who had a cesarean section at an early age in their previous pregnancies had to have a cesarean section again during the period conducted in the study. In addition, the rates of cesarean sections performed due to hypertensive diseases of pregnancy are similar between the groups.

Preterm delivery is a pregnancy complication whose cause is still not fully understood. Some researchers have emphasized that the high rate of preterm delivery in adolescents is due to inadequate prenatal care [17]. However, there are also studies in the literature that claim the opposite of this hypothesis. In their study, Chen and colleagues found that preterm birth rates were also common in women who received adequate prenatal care [18]. Some researchers have also emphasized that the reason why preterm delivery is more common in adolescent women may be due to adolescents not having sufficient gynecological maturity (short cervix or small uterine volume) [1, 19]. Karaçam and her colleagues find that in their meta-analysis, the rate of premature birth in pregnant adolescents is twice as high as in adults [20]. Contrary to some studies [1, 6, 9–11, 21], both groups have similar premature birth rates. In addition, the study results show that the birth weeks were similar in both groups. In addition, the study results show that birth weeks are similar in both groups.

According to the results of the study, the prevalence of pre-eclampsia is similar in both the adolescent and adult groups. Some studies in the literature describe similar results to the study [7, 22, 23]. Karaçam et al. Finds that the rates of pre-eclampsia, eclampsia, and gestational hypertension were similar between adolescents and adults in their meta-analysis study in Turkiye [20]. On the other hand, some studies reveal exactly the opposite results [24–26]. Studies indicating that pre-eclampsia rates are high in adolescents have reported that this may be due to inadequate prenatal care, low education, and socio-economic level.

According to the CDC, stillbirth is considered the death of a baby before or during birth. Stillbirth is also classified as early stillbirth (between 20 and 27 weeks of gestation), late stillbirth (between 28 and 36 weeks of gestation), and term stillbirth (after 37 weeks of gestation) [27]. Zhang and colleagues compared pregnant adolescents (10–19 years old) with pregnant adults (20–34 years old) in their study of 238,593 women in China between 2013 and 2017 and found that the risk of stillbirth and neonatal death was higher in the adolescent pregnancy group [24]. Similarly, a study conducted in Missouri reported that the risk of intrapartum stillbirth was higher in adolescent pregnant women than in adult pregnant women [28]. The groups according to stillbirth rates are compared in the study, but unlike the above studies, there is no statistical difference between the groups.

WHO defines low birth weight as a newborn weighing less than 2500 g. It also classifies the subgroups as very low birth weight (VLBW) (< 1500 g) and extremely low birth weight (ELBW) (< 1000 g). Marvin-Dowle et al. evaluated maternal and fetal outcomes of adolescent (≤ 19 years) and adult (20–34 years) pregnancies in primigravida women in Bradford in 2007–2010. The study results found that the rates of VLBW newborns were significantly higher in the adolescent group. They also reported that the rates of preterm birth were higher in the adolescent group [9]. In the study, the groups according to the average newborn birth weight are compared and found that the birth weight of the babies born in the adolescent group is lower than that of the adult group, which is statistically significant. This result is similar to the studies conducted [1, 9–11]. It is believed that this may be due to the fact that adolescents, who are already in the age of physiological, anatomical and psychological development, cannot adequately support the development of the fetus. In addition, socio-economic constraints also trigger the retardation of fetal development. Low birth weight is associated with prenatal and neonatal mortality and morbidity [29]. In addition, low birth weight newborns have a higher risk of developing chronic diseases in later years of life. Therefore, it is believed that it is important for pregnant adolescents to receive regular prenatal care, be educated about complications, and be provided with socio-economic support.

The Apgar score is the most important parameter to evaluate the newborn’s well-being. Doctor Virginia Apgar first defined it. It is evaluated in the first and fifth minutes after birth. The newborn’s skin color, heart rate, reflexes, muscle tone and respiration are evaluated by scoring 0, 1, and 2. A total of 7–10 points indicates that the newborn is likely well [30]. In addition, low Apgar scores have been reported to be associated with complications such as respiratory distress, hypothermia, and seizures [31]. Low fifth-minute Apgar values are associated with mortality and cerebral palsy [30]. In their retrospective cohort study, Yadav et al. compared adolescent and adult pregnant women according to Apgar scores and found no difference between the groups [19]. Ogawa and colleagues found that it was more common to give birth to babies with low Apgar scores in adolescents [32]. In the study, the groups are compared according to Apgar scores, and similar to the results of Yadav et al., no significant difference was found between the groups.

The most important limitation of this study is that it is a retrospective study by nature and that our study was conducted in a single center. The reason for the high adolescent pregnancy rates is that our hospital is a tertiary education and research hospital, and the majority of risky pregnancies are referred to our hospital. In addition, the good prenatal care and pregnancy follow-up of these patients in our hospital positively affect maternal and fetal outcomes.

## Conclusions

In conclusion, it can be assumed that adolescent pregnancies are vulnerable to complications, need prenatal support, and are at risk for socio-economic integration into society. The most important thing to do to reduce complications related to adolescent pregnancies should be to prevent them. Governments need to develop programs that are appropriate for their own social structures. It is believed that adolescents should be educated in schools on issues such as adolescent pregnancy complications, sexuality, and birth control methods.
